# Tailoring hydrophobic branch in polyzwitterionic resin for simultaneous capturing of Hg(II) and methylene blue with response surface optimization

**DOI:** 10.1038/s41598-017-04624-6

**Published:** 2017-07-04

**Authors:** Tawfik A. Saleh, Ihsan Budi Rachman, Shaikh A. Ali

**Affiliations:** Department of Chemistry, King Fahd University of Petroleum & Minerals, Dhahran, 31261 Saudi Arabia

## Abstract

A new highly efficient cross-linked polymer was synthesized via cyclotetrapolymerization of hydrophilic [(diallylamino)propyl]phosphonic acid hydrochloride (72 mol%), hydrophobic *N*,*N*-diallyl-1-[6-(biphenyl-4-yloxy)hexylammonium chloride (18 mol%), cross-linker 1,1,4,4-tetraallylpiperazinium dichloride (10 mol%) with an equivalent amount of alternating SO_2_ units (100 mol%). The pH-responsive resin chemically tailored with the aminopropylphosphonate chelating ligand and hydrophobic chain of (CH_2_)_6_OC_6_H_4_-C_6_H_5_ is designed to capture toxic metal ions and organic contaminants simultaneously. The developed resin was used for the remediation of Hg(II) ions and methylene blue from aqueous solutions as models. The experimental conditions were optimized utilizing the response surface methodology as an environmentally friendly method. The adsorption efficiency for Hg(II) was ≈100% at 10 ppm initial concentration at pH 5 at 25 °C, while it was 80% for removal of the dye in a single pollutant system. Interestingly, the resin demonstrated its remarkable efficacy in the simultaneous and complete removal of Hg(II) and the dye from their mixture. Increased removal of the dye (≈100%) in the presence of Hg(II) was attributed to the synergistic effect. The equilibrium data were evaluated by employing the Langmuir and Freundlich isotherm models.

## Introduction

Heavy metal ions are non-biodegradable pollutants which accumulate in groundwater and on the soil surface as a waste of industrial processes such as minerals mining, pigments for color enhancement, and anti-corrosion additive materials coating. Heavy metal ions over the normal limit can be toxic and may cause a variety of diseases, including loss of memory, kidney and renal problems, diarrhea, lung damage, cancer, as well as reproductive disorders. Mercury is the most dangerous metal ion due to its toxicity^1, 2^.

Other types of pollutants are the organic compounds having adverse effects on the environment. Dyes such as those used in textiles, paper painting, leather, plastics, rubber and dying are widely used in industry^3, 4^. The disposal of dyes from the textile industry leads to serious environmental damage owing to their difficult degradability. 3,7-bis(dimethylamino) phenothiazine-5-ium-chloride (otherwise known as methylene blue (MB)) is one example of the many dyes used extensively in industry; it can be absorbed by plants, and consequently consumed by humans and accumulated in human tissue, leading to many diseases in humans owing to its non-biodegradability and toxicity.

To minimize the impact on the environment, there are many methods for the removal of pollutants, like membrane processes, continuous liquid-solid separation, liquid extraction, ion exchange, filtration, electrolytic recovery, reverse osmosis, advanced oxidation processes, adsorption, chemical precipitation as sulfides, hydroxides or carbonates, and adsorption with ultrasonically assisted acid treatment^5^. Among these, the adsorption methods have become significant because of their efficiency, simplicity, low cost, and usefulness at low concentrations. Until now, numerous publications have dealt with the removal of a single pollutant, while a few studies have reported the simultaneous removal of organic and metal pollutants by adsorption techniques^6, 7^.

Choosing an appropriate and efficient adsorbent material for a specific pollutant target should be the first priority for a successful adsorption process. Conventional adsorbents like clay activated carbon and nanomaterials are usually used for the removal of heavy metal ions and dyes from wastewater^8^. Non-toxic, biodegradable and eco-friendly natural polymers such as cellulose, chitosan, and starch are also used because of their natural abundance, high efficiency and low cost^9^. For industrial applications, there is a need for more advanced properties of an adsorbent such as fast adsorption kinetics, high capacity, and temperature stability^10–15^. Polymeric materials can fulfill the industrial requirements by tailoring them with judicious design.

**Figure 1:**
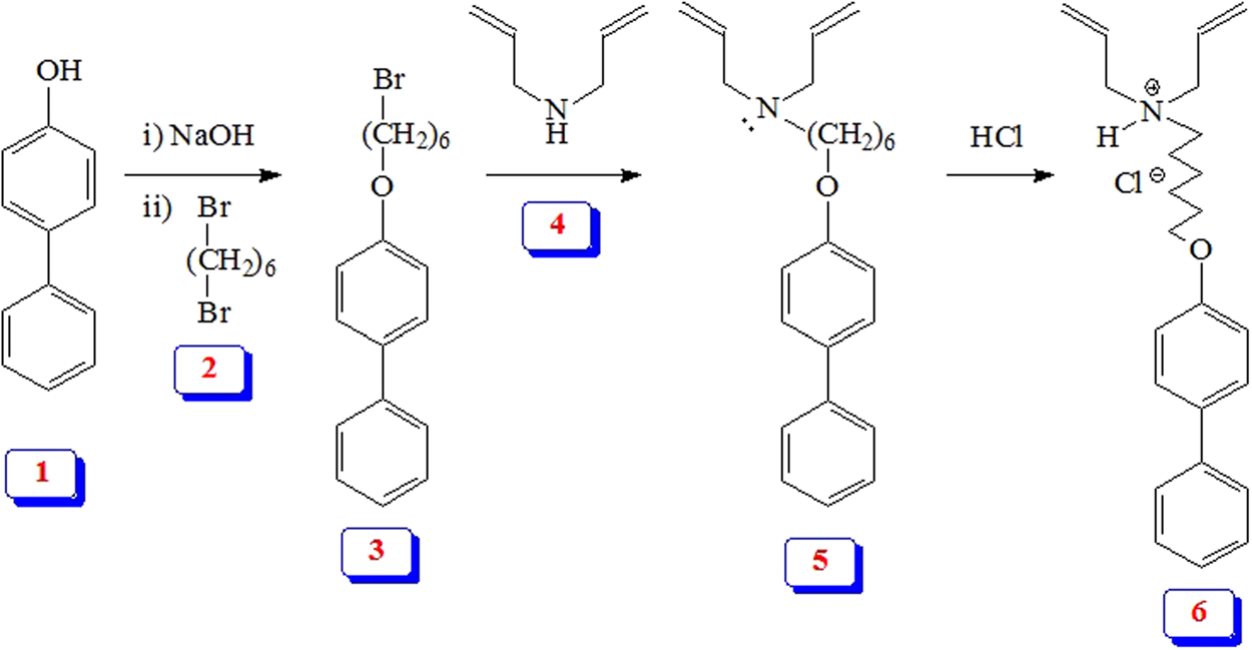
Synthesis of hydrophobic monomer **6**.

**Figure 2:**
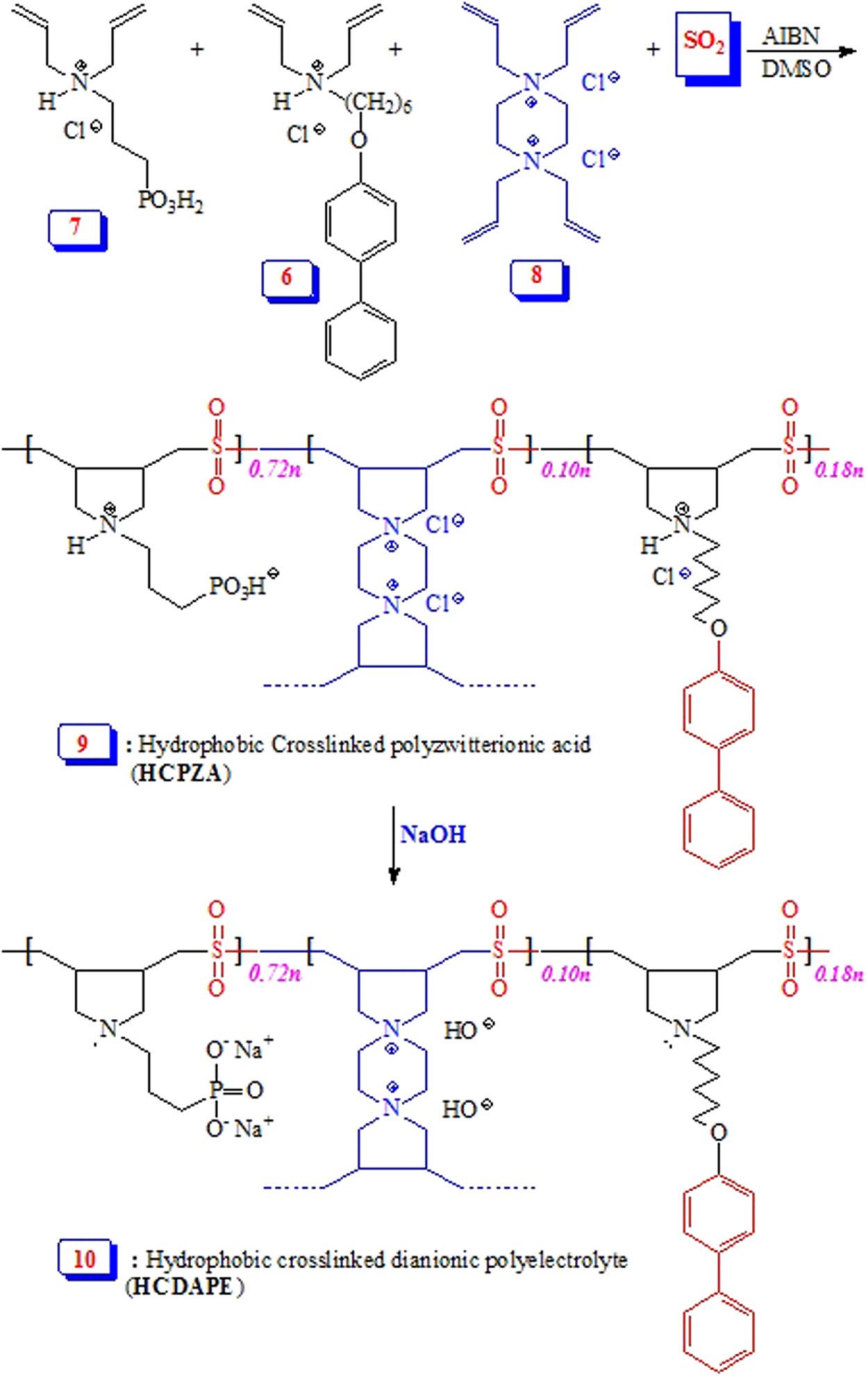
Synthesis of a hydrophobic cross-linked polyzwitterion/anion resins.

The current work describes the synthesis of a novel functionalized resin embedded with the residues of the chelating ligand of aminopropyl phosphonate and hydrophobic pendants (Figs 1 and 2). The resin could be used as a sorbent for the simultaneous removal of Hg(II) ions and methylene blue from aqueous solution.

## Experimental

### Materials

2,2′-Azoisobutyronitrile (AIBN), purchased from Fluka AG, was purified by crystallizing from a chloroform-ethanol mixture. Dimethyl sulfoxide (DMSO) was purified by drying (CaH_2_) and distilling at 64–65 °C (4 mmHg). *p*-Phenylphenol (**1**), 1,6-dibromohexane (**2**) and diallylamine (**4**) were purchased from Fluka AG. Cross-linker **8** was synthesized using a literature procedure^16^. Monomer **7** and 4-(6-Bromohexyloxy)biphenyl (**3**) were prepared as described earlier^17^.

An Hg(NO_3_)_2_ standard solution (1000 ppm) was used to prepare the diluted solutions of the required concentrations. Sodium hydroxide and nitric acid were purchased from Sigma–Aldrich. Millipore water (18.2 MΩ·cm) was used for the adsorption study. Reagent grade organic solvents were used.

### Characterization Techniques and procedures

A Perkin Elmer 2400 Series II CHNS/O Elemental Analyzer was used for the elemental analysis. A Perkin Elmer Elemental Analyzer Series 11 Model 2400 (Waltham, Massachusetts, USA) was used for elemental analysis, while the IR analyses were performed on a Thermo scientific FTIR spectrometer (Nicolet 6700, Thermo Electron Corporation, Madison, WI, USA). NMR spectra were obtained in a JEOL LA 500 MHz spectrometer using CDCl_3_ with tetramethylsilane (TMS) as an internal standard (^1^H signal at δ0 ppm), while taking the HOD signal at δ4.65 ppm and the dioxane signal at 67.4 ppm as internal and external standards in D_2_O, respectively. The solid-state CP MAS ^13^C NMR spectrum was recorded on a Bruker 400 MHz NMR Spectrometer using a spin rate of 4000 Hz, using a chemical shift of CH_2_ of adamentane at 29.5 ppm as an external standard^18^.

A scanning electron microscope (SEM) was used to examine the morphology of the polymer surface before and after the adsorption of Hg(II). Energy-dispersive X-ray spectroscopy (EDX) fitted with a detector model X-Max was used to obtain the elemental spectrum and obtain elemental analyses of the original polymer and the pollutant-loaded resin. A thermogravimetric analysis (TGA) using an SDT Q600 thermal analyzer from TA instruments, USA, was conducted to evaluate the thermal stability of the prepared resin. The temperature was raised at a constant rate of 10 °C/min over a temperature range of 20–800 °C in an air atmosphere flowing at a rate of 100 mL/min. The specific surface area and pore size distribution were determined by using the methods of Brunauer-Emmett-Teller (BET) and Barrett-Joyner-Halenda (BJH). A mercury analyzer was employed to monitor the concentration of Hg(II). The concentration of the tested dye was monitored in an UV-vis spectrophotometer using optical quartz cuvettes.

### Synthesis of monomer precursor 5

A solution of bromide **3** (4.60 g, 13.8 mmol) and diallylamine **4** (6.7 g, 69 mmol) in toluene (6 mL) was heated under N_2_ at 100 °C for 24 h. The reaction mixture was taken in water 20 (mL) containing NaOH (0.60 g, 15 mmol) and extracted with ether (2 × 25 mL). The organic extract was dried (Na_2_SO_4_) and concentrated. The residual liquid was purified by chromatography over silica gel using an ether/hexane mixture as eluent to obtain amine **5** (4.04 g, 84%) as a colorless liquid.

(Found: C, 82.2; H, 8.8; N, 3.9%. C_24_H_31_NO requires C, 82.48; H, 8.94; N, 4.01). ν_max._ (neat) 3075, 3030, 3004, 2972, 2937, 2858, 2796, 1609, 1519, 1488, 1416, 1388, 1290, 1268, 1246, 1178, 1075, 1046, 996, 918, 833, 762, and 697 cm^−1^; δ_H_ (CDCl_3_) 1.35 (2H, quint, *J* 7.4 Hz), 1.48 (4H, m), 1.80 (2H, quint, *J* 7.0 Hz), 2.43 (2H, t, *J* 7.3 Hz), 3.09 (4H, d, *J* 5.8 Hz), 3.98 (2H, t, *J* 6.6 Hz), 5.14 (4 H, m), 5.86 (2H, m), 6.95 (2H, d, *J* 8.8 Hz), 7.29 (1 H, t, *J* 8.0 Hz), 7.40 (2H, t, *J* 7.5 Hz), 7.51 (2H, d, *J* 8.8 Hz), 7.54 (2H, d, *J* 7.3 Hz); δ_C_ (CDCl_3_) 26.02, 26.87, 27.25, 29.27, 53.23, 56.88 (2C), 67.96, 114.74 (2C), 117.30 (2C), 126.58, 126.69 (2C), 128.09 (2C), 128.68 (2C), 133.53, 135.82 (2C), 140.86, 158.67 (TMS: 0.00 ppm). DEPT 135 NMR analysis supported the ^13^C spectral assignments.

### Synthesis of monomer 6

Dry HCl was passed through a solution of **5** (1.90 g, 5.436 mmol) in ether (30 mL) at 0–5 °C. The precipitated white salt was filtered and washed with ether to obtain **6** (2.01 g, 96%).

M.p. 108–110 °C; (Found: C, 74.4; H, 8.4; N, 3.6%. C_24_H_32_ClNO requires C, 74.68; H, 8.36; N, 3.63). ν_max._ (KBr) 3478, 3402, 3327, 3224, 3084, 3029, 2946, 2867, 1653, 1623, 1606, 1518, 1488, 1392, 1289, 1270, 1244, 1194, 1176, 1116, 1043, 1021, 997, 944, 848, 821, 772, 720, and 701 cm^−1^; δ_H_ (CDCl_3_) 1.42 (2H, quint, *J* 7.3 Hz), 1.53 (2H, quint, *J* 7.3 Hz), 1.80 (2H, quint, *J* 6.7 Hz), 1.89 (2H, m), 2.96 (2H, m), 3.63 (4H, m), 3.98 (2H, t, *J* 6.4 Hz), 5.52 (4H, m), 6.14 (2H, m), 6.95 (2H, d, *J* 8.6 Hz), 7.29 (1H, t, *J* 8.3 Hz), 7.41 (2H, t, *J* 7.6 Hz), 7.52 (2H, d, *J* 8.8 Hz), 7.54 (2H, d, *J* 8.3 Hz), 12.48 (1H, s); δ_C_ (CDCl_3_) 23.35, 25.53, 26.53, 28.96, 51.66, 54.63 (2C), 67.53, 114.73 (2C), 125.66 (3C), 126.26, 126.63 (2C), 128.09 (2C), 128.70 (2C), 133.62, 140.72 (2C), 158.50 (TMS: 0.00 ppm). DEPT 135 NMR analysis supported the ^13^C spectral assignments.

### Synthesis of the resin

#### Tetrapolymerization of monomers 6, 7, cross-linker 8 and SO_2_ to hydrophobic cross-linked polyzwitterionic acid (HCPZA) 9

To a solution of **7** (5.10 g, 20 mmol), **6** (1.93 g, 5 mmol), and **8** (0.890 g, 2.78 mmol) in dimethyl sulfoxide (12.8 g) in a round bottom flask (50 cm^3^) was absorbed SO_2_ (1.96 g, 30.6 mmol). Initiator AIBN (250 mg) was added under N_2_, then the mixture was stirred in the closed flask at 65 °C. Within 1 h, the mixture became an immovable gel. More DMSO (6.2 g) was added, and the polymerization was continued at 67 °C for 24 h. A few times the flask was cooled and opened to release the produced N_2_. Then, the obtained transparent gel was soaked in water; the white resin was repeatedly washed with water and finally with acetone. Resin HCPZA **9** was dried under vacuum at 65 °C for 6 h to a constant weight (8.55 g, 87%). The composition of the resin was found to be: C, 43.0; H, 6.8; N, 4.5; S, 10.5. The incorporated monomers in HCPZA **9** containing **7** (72.0 mol%), **6** (18.0 mol%), **8** (10.0 mol%) and SO_2_ (100 mol%) requires C, 43.39; H, 6.61; N, 4.72; S, 10.82%. ν_max._ (KBr) 3445 (br), 2920, 2854, 1635, 1518, 1483, 1467, 1410, 1310, 1249, 1128, 1029, 903, 831, 764, 699, 596, and 510 cm^−1^.

#### Conversion of HCPZA 9 to hydrophobic cross-linked dianionic polyelectrolyte (HCDAPE) 10

Resin **9** (1.00 g, 3.0 mmol) was treated with NaOH (0.24 g, 6.0 mmol) in water (20 mL); after 1 h at room temperature, a mixture of methanol (50 mL) containing NaOH (0.12 g, 3.0 mmol) was added to the gel. The resultant HCDAPE **5** was then filtered, washed with methanol and dried under vacuum for 6 h at 65 °C (1.05 g, 99%).

The composition of the resin was found to be: C, 40.7; H, 5.9; N, 4.2; S, 9.6. The incorporated monomers as in HCDAP **10** containing repeating units derived from **7** (72.0 mol%), **6** (18.0 mol%), **8** (10.0 mol%) and SO_2_ (100 mol%) requires C, 41.07; H, 5.73; N, 4.35; S, 9.96%. ν_max._ (KBr) 3430, 2924, 2784, 1667, 1611, 1521, 1487, 1416, 1308, 1245, 1182, 1125, 1049, 972, 834, 765, 695, 552, and 499 cm^−1^.

### Swelling coefficient

The swelling coefficient was evaluated as follows: resins were crushed and a 20- to 30-mesh fraction was used. The coefficient of swelling is defined as the ratio of the settled wet volume of the resin to the dry volume. Thus, to a known volume of the dry resin in a burette, sufficient water was added to cover it and the volume of the wet resin was measured after no further change in the volume occurred, and compared with the volume of the dry resin.

### Adsorption experiment

Adsorption experiments were performed using solutions with a known concentration of methylene blue, mercury(II), or a mixture of both in a container containing a predetermined amount of the resin and placed on a shaker at a predetermined rpm speed. The pH of the solution was adjusted with HNO_3_ and NaOH solutions using a pH meter. The dye concentration was determined by a UV–Vis spectrophotometer while the concentration of the Hg(II) was monitored using a mercury analyser. The experiments were executed at room temperature (25 °C). The influence of the parameters (i.e. pH, dosage, initial concentration, shaking speed and temperature) controlling the adsorption were optimized using a central composite design (CCD) which is the most popular response surface method (RSM) design. This approach helps us to investigate the effect of parameters in a cost- and time-effective way consuming less material so as to be environmentally friendly.

### Kinetic and Isotherm studies

The kinetic studies were performed under the optimum conditions of shaking speed (150 rpm) and pH (5). Containers containing a mixture of the adsorbates and resin were agitated in a shaker at 25 °C and aliquots were collected at time intervals of 2, 5, 10, 20, 30, 40, 50, 60, 70, 80, 90 and 120 mins. After the equilibrium, a final methylene blue concentration was analyzed using a UV–Vis spectrophotometer and the Hg concentration was analyzed by a mercury analyzer. The adsorption capacity for (*q*_e_) was calculated using the equation:

1 $${q}_{{\rm{e}}}=({C}_{{\rm{o}}}-{C}_{{\rm{e}}})\times \frac{V}{m}$$

where *V* stands for the volume of the solutions (mL) and *m* is the mass in gram (g) of adsorbent. All the experiments were performed in duplicate and the relative standard deviation was lower than 3.0%. The values of kinetic and isotherm parameters were determined by a linear regression analysis.

## Results and Discussion

### Characterization of the polymer

**Figure 3:**
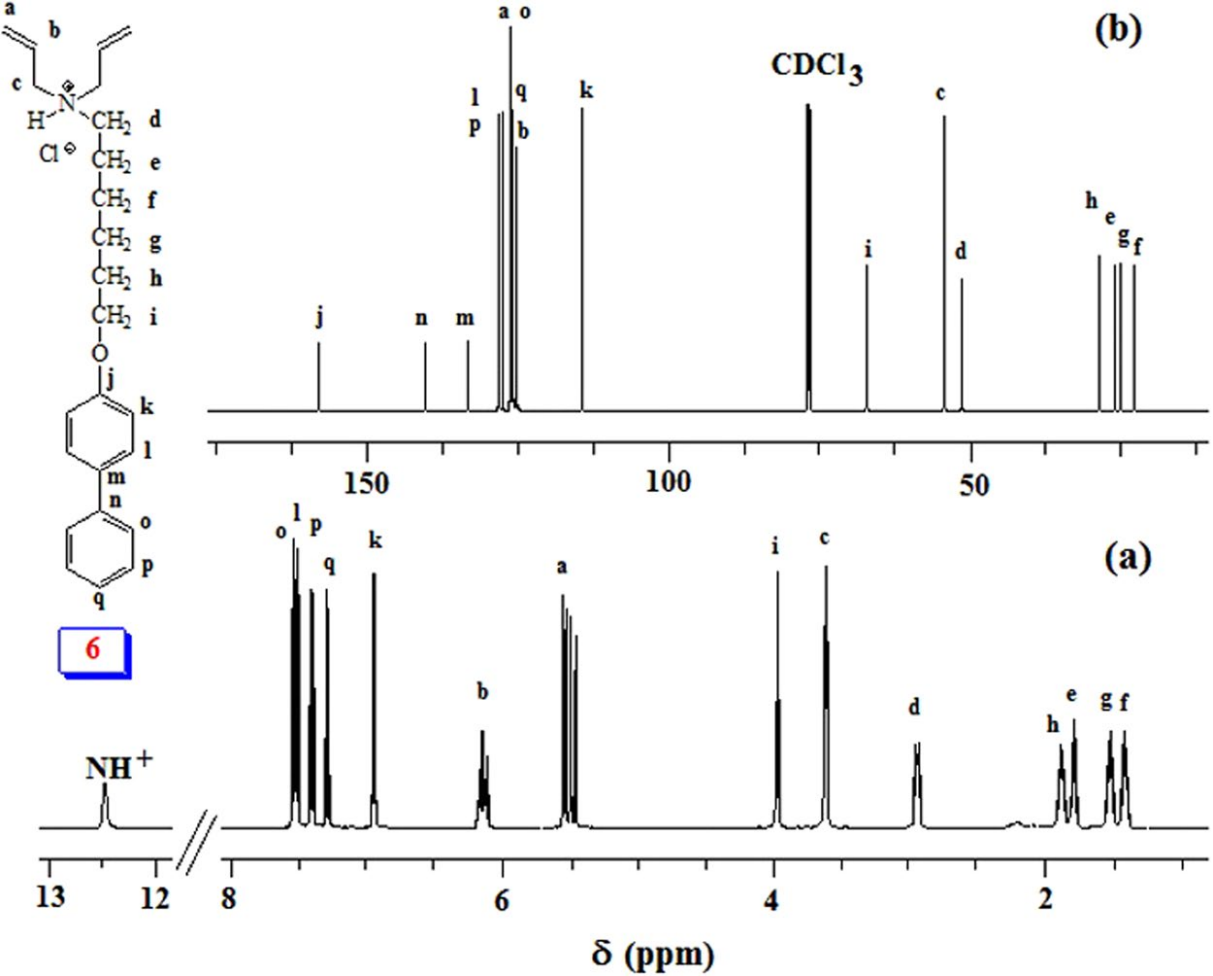
^1^H and ^13^C NMR spectra of monomer **6**.

A new hydrophobic monomer **6** was synthesized as outlined in Fig. 1 in excellent yield. Thus, *p*-Phenylphenol (**1**) was alkylated with 1,6-dibromohexane (**2**) to **3** which on treatment with diallyl amine (**4**) afforded monomer precursor **5** in 84% yield. Monomer **6** was then obtained by treating **5** with gaseous HCl in 96% yield. The monomer was characterized by elemental and spectral analyses: ^1^H and ^13^C NMR spectra (Fig. 3) confirmed its structure. The chemical shifts were assigned based on substituent effects; the spectrum revealed each and every carbon signal (Fig. 3b).

**Figure 4:**
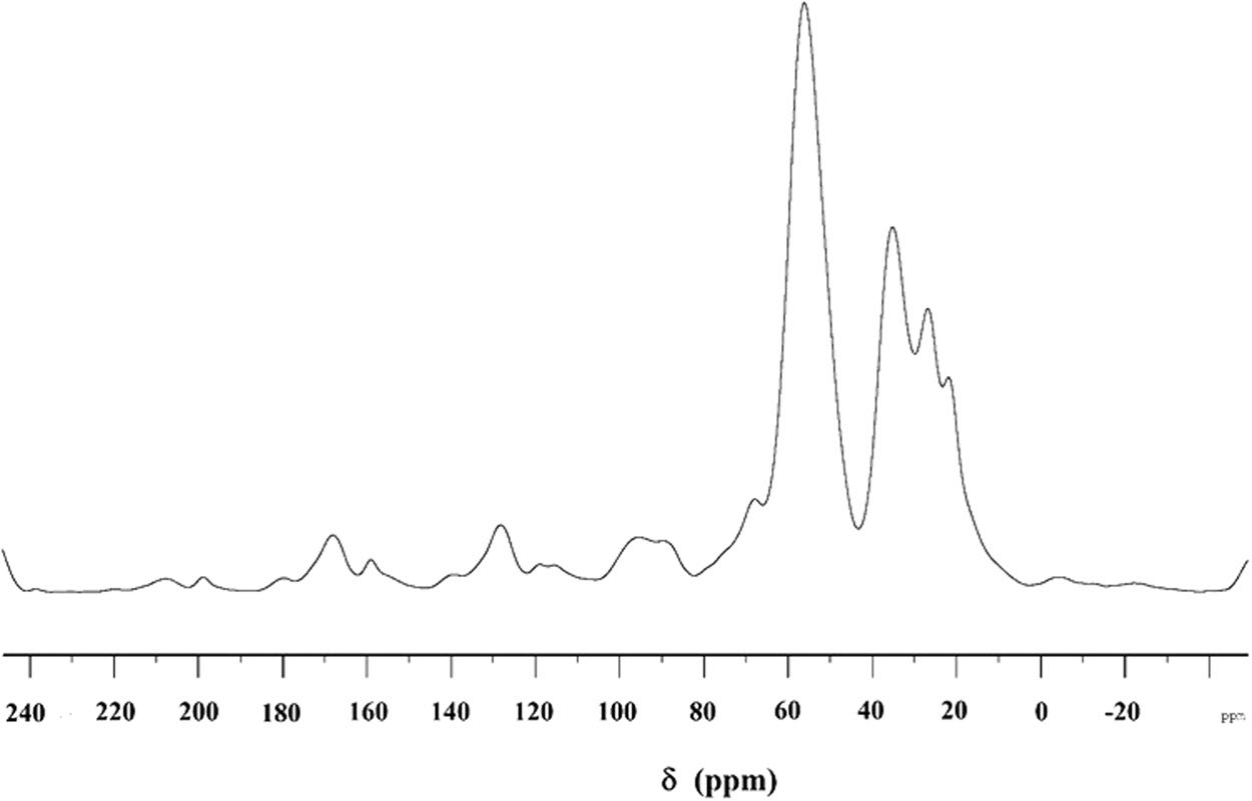
^13^C solid NMR of HCPZA **9**.

Butler’s cyclopolymerization protocol^19–22^ was exploited in the AIBN-initiated tetrapolymerization of hydrophobic monomer **6**, hydrophilic monomers **7** and cross-linker **8**, along with alternating SO_2_ as the fourth monomer to obtain hydrophobic cross-linked polyzwitterionic acid (HCPZA) **9** in 87% yield. During the work up, HCl is eliminated to give the zwitterionic aminophosphonate motifs. The composition of the repeating units in the resin matched with the feed ratio of 0.18:0.72:0.10:1.0 for monomers **6**/**7**/**8**/SO_2_ as supported by elemental analysis: this is expected for such a high degree of conversion to the resin (in 87% yield). The incorporation of hydrophobic monomer was confirmed by a solid-state ^13^C NMR spectrum which revealed the presence of carbon signals in the aromatic region (110–150 ppm) (Fig. 4).

HCPZA **9** upon treatment with NaOH was converted to HCDAPE **10**. Zwitterionic resin **9** and its anionic form **10** were found to have swelling coefficients of 2.2 and 5.8, respectively. The zwitterionic form in a compact coil is expected to have a lower affinity for adsorption of water, while anionic form **10** has more expanded conformations owing to the repulsion between negative charges and thus has a greater affinity for solvation.

Note that the incorporation of the hydrophobic monomer into the cross-linked polymer would serve dual purposes: the simultaneous removal of toxic metals, as well as organic contaminants, from wastewater. In a single treatment, it would exploit the chelating ability of the aminophosphonate ligand to capture metal ions and the hydrophobic surface of the long chain hydrocarbons to scoop up the organic contaminants. We did anticipate an exciting outcome.

**Figure 5:**
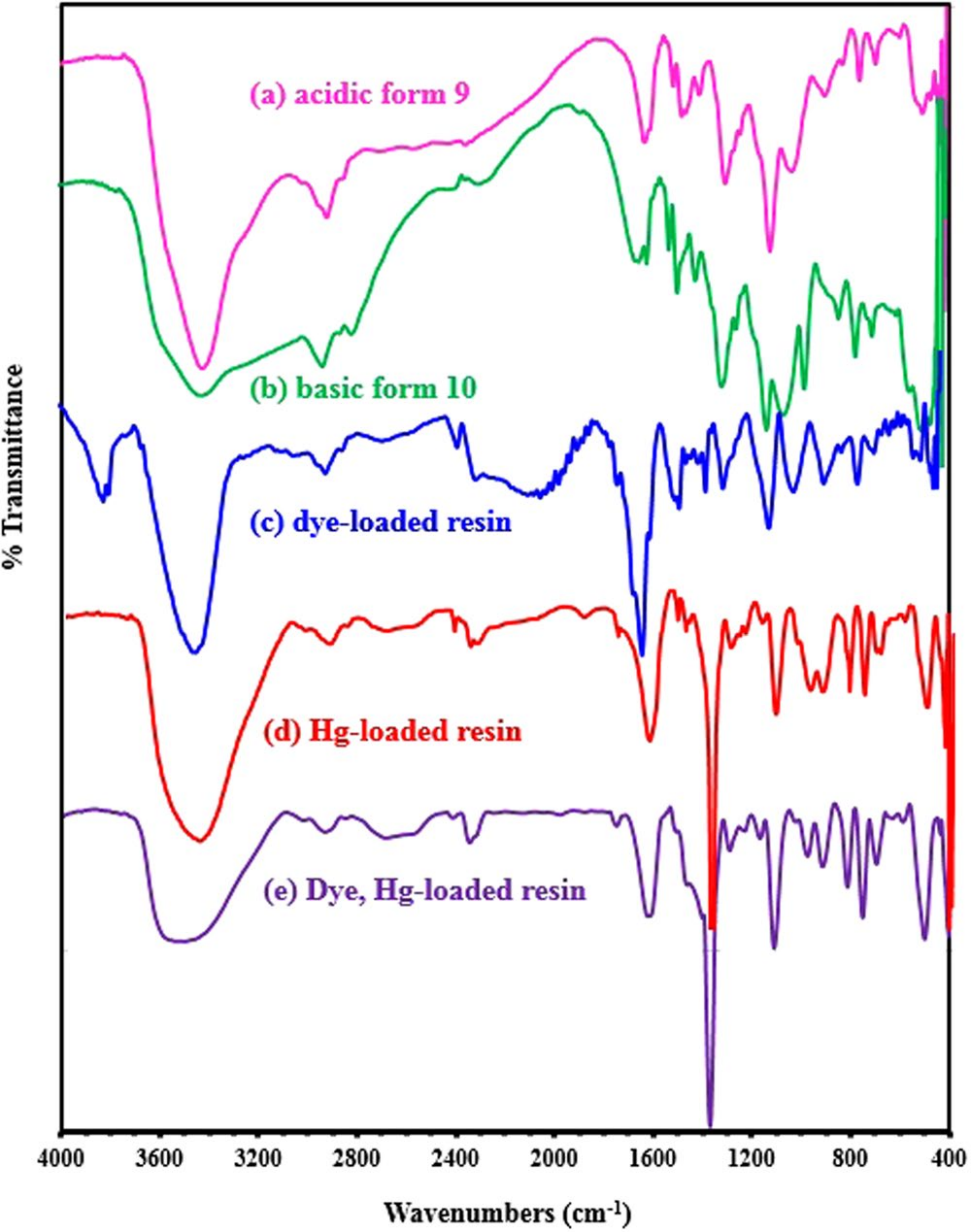
IR Spectra of the cross-linked resin (**a**) in acidic form **9** and (**b**) in basic form **10**. IR Spectra of (**c**) dye-loaded resin **9**, (**d**) Hg-loaded resin **9** and (**e**) Dye, Hg-loaded resin **9**.

The IR spectrum was obtained for the polymer in acid and basic forms as depicted in Fig. 5. The spectra show bands at ≈1310 cm^−1^ and ≈1125 cm^−1^ which can be assigned to the asymmetric and symmetric bands of SO_2_^23^. Phosphonate groups display intensive IR absorption bands at 560–600 cm^−1^ and 1000–1100 cm^−1^ regions. The adsorbed water band can be seen at around 3400 cm^−1^ of the resin in acidic form **9**, and at the relatively wide range of from 2600 to 3600 cm^−1^; in basic form **10**, the resin displayed an explicit peak at 3470 cm^−1^ while a weaker peak is also found at 630 cm^−1^. The bands at around 1600–1680 cm^−1^ can be assigned to the C=C stretching.

**Figure 6:**
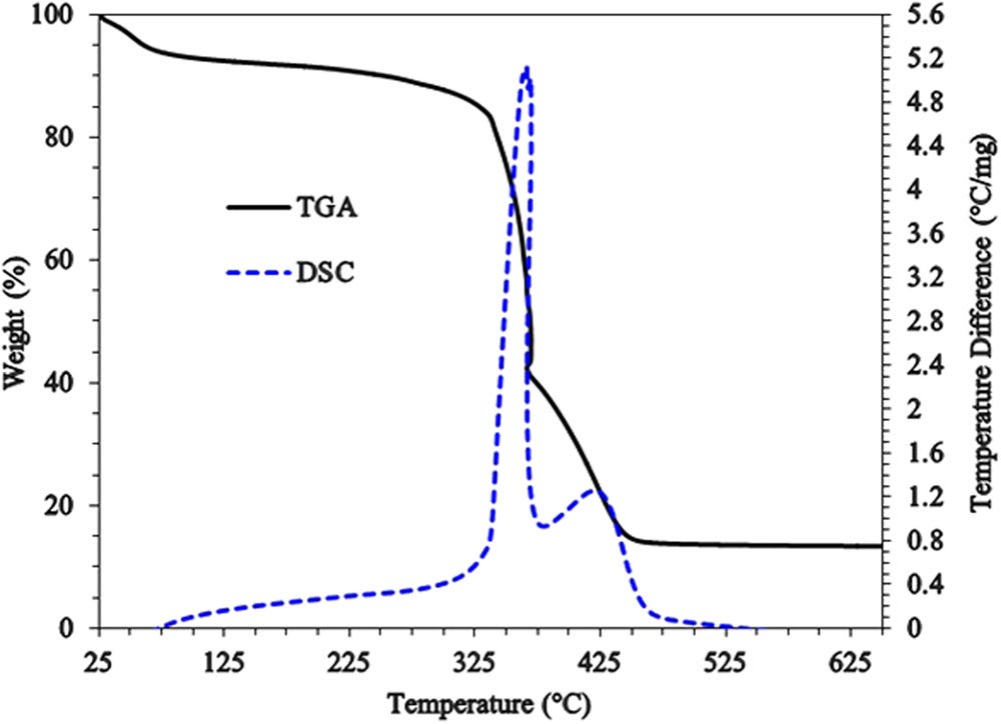
TGA curves of HCPZA **9**.

The TGA curve of **9**, shown in Fig. 6, revealed two distinct weight loss steps. The first slow but gradual weight loss of about 13% is attributed to the removal of moisture and water molecules embedded in the cross-linked polymer. The second dramatic loss of about 70% around 335 °C is attributed to the loss of phosphonate pendants and SO_2_ owing to polymer degradation. It could also be attributed to the combustion of nitrogenous organics with the release of NOx, CO_2_, and H_2_O gasses^23^. The polymer remained stable even at 250 °C. The DSC curve indicates that there is a crystalline melt defined by the peak temperature at around 350 °C.

### Results of the factorial design

The central composite design (CCD), which is the most popular response surface method (RSM), was implemented to design a series of tests with the least number of experiments. This approach helped us to investigate the effect of the parameters involved (i.e. pH, dosage, initial concentration, shaking speed and temperature) on the responses (i.e. the removal percentage) in a cost- and time-effective way. It also helped to consume less material to make the process environmentally friendly. The CCD makes it feasible to observe the possible interaction of the parameters and their influences on the removal efficiency. The experimental factors that have been included are pH (3–7), adsorbent dosage (10–30 mg), initial concentrations (0.1–10 µM dye and 10–70 ppm Hg(II)), shaking speed (50–150 rpm) and temperature (25–65 °C) with a 95% confidence limit. The design of experiment (DOE) is considered to be more informative than the one-variable-at-a-time experimental procedures since it gives an indication of the interaction between the factors. The low and high levels of the initial Hg(II) concentrations were selected to simulate the real industrial wastewaters. The type of design was 2-level factorial (default generators) (Table 1). Based on the experimental design, the adsorption tests were performed using adsorbent **9**. The percentage removal of the Hg or dye under certain conditions is the response variable in this study.

**Table 1:** Design matrix of the factorial design in the central composite design (CCD).

Variable	Low (−)	Central point (0)	High (+)
Parameters optimization for dye removal			
pH	3	5	7
Adsorbent dosage (mg)	10	20	30
Initail concentration (µM)	0.1	5.05	10
Shaking speed (rpm)	50	100	150
Temperature (°C)	25	45	65
Parameters optimization for Hg removal			
pH	3	5	7
Adsorbent dosage (mg)	10	20	30
Initail concentration (ppm)	10	40	70
Shaking speed (rpm)	50	100	150
Temperature (K)	25	45	65

**Figure 7:**
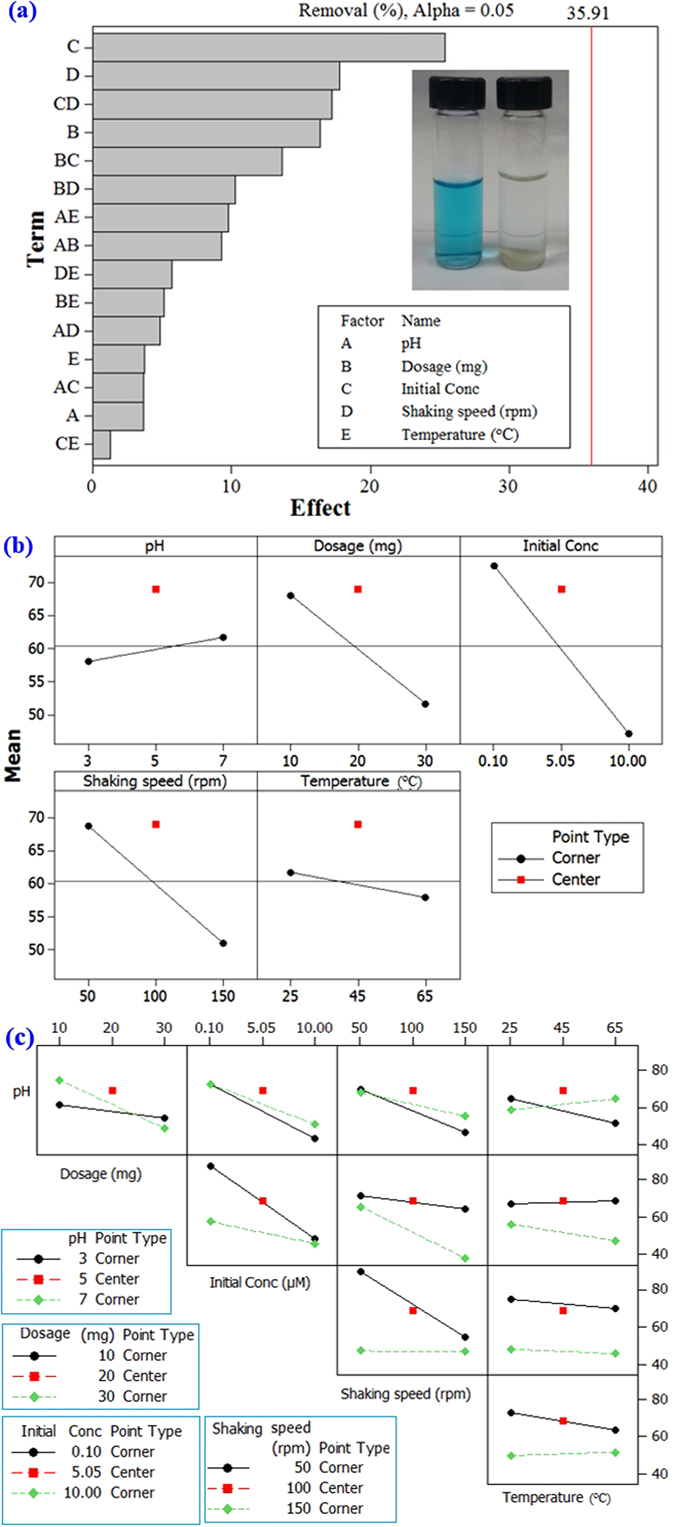
The factorial design for the optimization of adsorption of dye showing: (**a**) the Pareto chart, (**b**) the half-normal plot of the effects; (**c**) the factorial design showing the interaction plot.

The factorial design plots, including the Pareto chart and the normal plot of the effects, are depicted in Fig. 7. The Pareto chart indicates that the most significant factors influencing the adsorption of dye on the prepared adsorbent are initial concentration, shaking speed and dosage. The half-normal plot of the effects indicates that the removal of dye increases with increasing the pH of the solution, while at low dye concentration the removal percentage was high. There was a decrease in the removal due to raising the temperature, though the change was relatively insignificant. The interaction plot for the response shown in Fig. 7c indicates that the interaction between the dosage, initial concentration and shaking speed have the highest effect on the adsorption of dye. The interaction between the media pH and the initial concentration was also significant in affecting the removal efficiency.

**Figure 8:**
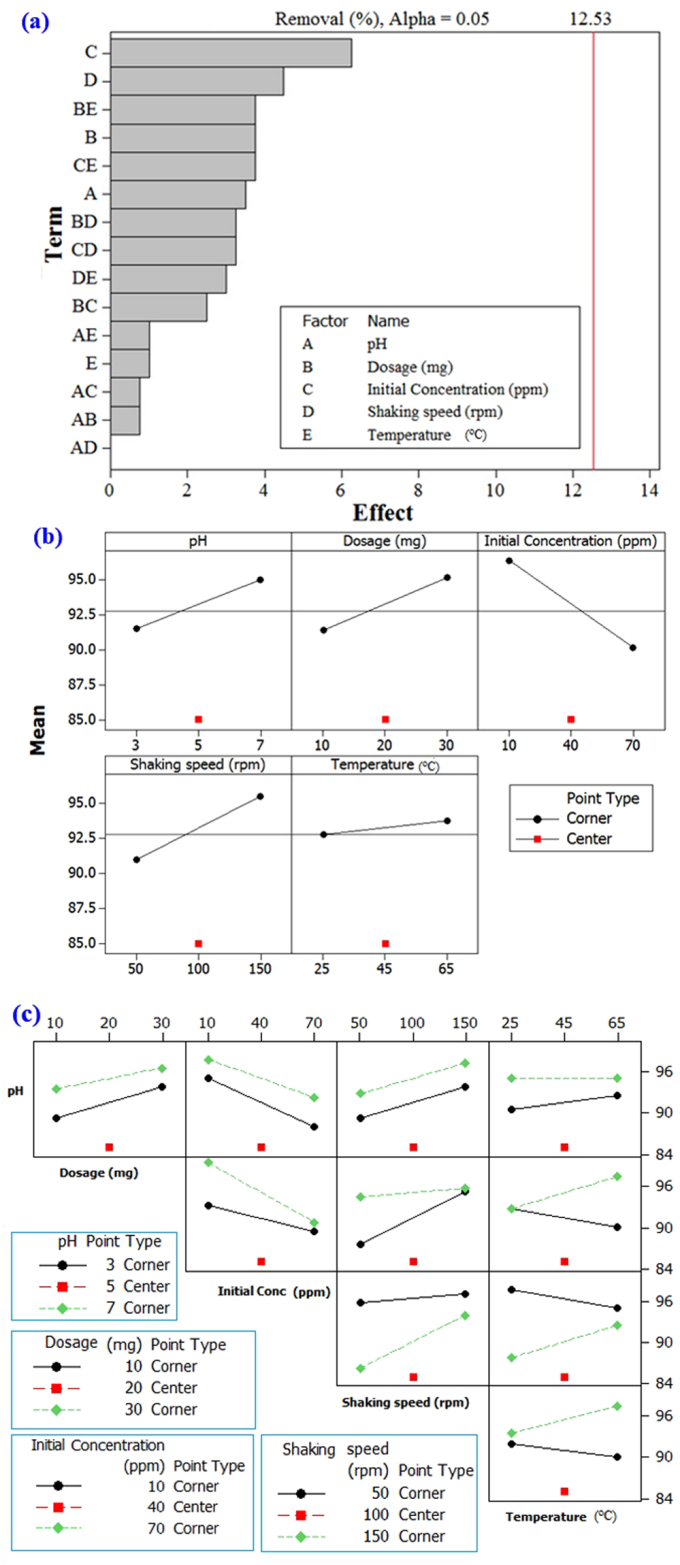
The factorial design for the optimization of the adsorption of Hg(II) showing: (**a**) the Pareto chart, (**b**) the half-normal plot of the effects; (**c**) the factorial design showing the interaction plot for a response.

Considering the optimization of parameters for the Hg(II) adsorption, the Pareto chart shown in Fig. 8a indicates that the most significant factors influencing the adsorption of Hg(II) on the resin are the initial concentration, temperature and shaking speed. The half-normal plot of the effects indicates that the highest removal was obtained at a high pH (studied in the range 3–7, Table 1) along with increasing the shaking speed. By increasing the temperature, the removal was enhanced. The interaction plot for the response shown in Fig. 8c indicates that the highest interactions between the experimental factors were the interaction between the temperature and dosage, followed by the interaction between the initial concentration and the temperature.

### Adsorption kinetics

Lagergren’s first-order and pseudo-second-order kinetics models were implemented to examine the controlling mechanism of mercury and methylene blue adsorption from aqueous solutions. Adsorption equilibrium was reached in 40 mins. The linear equation for Lagergren’s first order kinetics is given as^24^:

2 $$\mathrm{ln}({q}_{e}-{q}_{t})=\,\mathrm{ln}\,{q}_{e}-\,{k}_{1}t$$

First order kinetics using *q*_t_ with contact time for the removal of the dye and Hg by the resin for different feed concentrations; indicates that the experimental data do not fit with the first order model (Table 2). Therefore, the experimental data were evaluated using the pseudo-second adsorption kinetic rate equation^25^:

3 $$\frac{d{q}_{t}}{dt}={k}_{2}{({q}_{e}-{q}_{t})}^{2}$$

*k*_2_ depicted the rate constant of the pseudo-second order adsorption (g/mg.min), *q*_e_ and *q*_t_ are the adsorbed amount (capacity) of Hg(II) at equilibrium and at time *t*.

**Table 2:** Adsorption kinetic parameters for Lagergren models.

Adsorbate	*q*_e,exp_ (mg g^−1^)	Lagergren’s first order			Pseudo-second order			
		*k*_1_ (min^−1^)	*q*_e,cal_ (mg g^−1^)	*R* ^*2*^	*q*_e,cal_ (mg g^−1^)	*k*_2_ (g mg^−1 ^min^−1^)	H^*a*^	*R* ^*2*^
3.2 ppm MB^b^	2.54	0.04	8.25	0.8542	2.86	0.61	1.44	0.9980
3.2 ppm MB mixed with 10 ppm Hg (II)	2.85	0.03	6.01	0.8774	2.95	0.25	1.56	0.9971
0.32 ppm MB^c^	1.95	0.06	4.08	0.7692	2.10	0.23	1.92	0.9968
0.32 ppm of MB mixed with 10 ppm Hg (II)	2.03	0.07	3.98	0.9807	2.05	0.35	1.23	0.9965
10 ppm Hg	7.01	0.05	5.25	0.8835	7.31	0.36	1.63	0.9989
10 ppm Hg mixed with 3.2 ppm MB	7.85	0.06	6.85	0.9781	8.10	0.72	1.01	0.9988
70 ppm Hg	55.3	0.09	8.84	0.9879	56.01	0.62	1.27	0.9991
70 ppm Hg mixed with 3.2 ppm MB	58.2	0.08	7.52	0.8968	57.85	0.65	1.48	0.9958

^*a*^Initial adsorption rate, h = k_2_q_e_^2^.

^b^i.e. 1 × 10^−5^ M of MB.

^c^i.e. 1 × 10^−6^ M of MB.

The linear form of the pseudo-second-order is written as:

4 $$\frac{t}{{q}_{t}}=\,\frac{1}{\,{k}_{2}{q}_{e}^{2}}+\,\frac{t}{{q}_{e}\,}$$

where *q*_e_ and *q*_t_ are the adsorption capacities at equilibrium and at time *t* (min) respectively. *k*_2_ (g/(mg.min)) is the pseudo-second-order rate constant. The values of *q*_e_ and *k*_2_ can be calculated from the slope and intercept of the *t*/*q*_t_ versus *t* plot. The results listed in Table 2 show a high correlation coefficient (*R* ^*2*^ ≥ 0.99), which suggests that the kinetics data were better described by a pseudo-second-order kinetics model. The adsorption capacity was determined as a function of the adsorbate initial concentration and adsorbent dosages. The calculated equilibrium adsorption capacities were consistent with the obtained experimental results. Screening the literature, it was found that the equilibrium adsorption capacities of methylene blue and Hg on the current adsorbent were relatively close to that found on various adsorbents reported in the literature, considering low initial concentrations ranges^8, 9, 12, 15^.

### Adsorption isotherms

It is imperative to describe the mechanism associated with the adsorption process by which the adsorbate is adsorbed. The equilibrium data of dye, Hg and the mixture of both were evaluated by the Langmuir and Freundlich isotherms. The Langmuir isotherm postulates a monolayer adsorption which takes place at the binding sites with no interactions between the molecules adsorbed, in addition, transmigration on the surface of the sorbent does not take place. The Langmuir equation is given by Equation (5)

5 $$\frac{{C}_{e}}{{q}_{e}}=\frac{1}{{k}_{L}{q}_{m}}+\,\frac{{C}_{e}}{{q}_{m}}$$

where *q*_m_ (mg/g) is the quantity of monolayer adsorbate required to form a single monolayer on a unit mass of the adsorbent (mg**·**g^−1^), *q*_e_ is the amount adsorbed on a unit mass of adsorbent (mg**·**g^−1^) at equilibrium concentration *C*_e_ (mg**·**L^−1^) and *k*_L_ is the Langmuir equilibrium constant (L**·**mg^−1^) that takes care of the apparent energy of adsorption. A plot of C_e_/*q*_e_ against C_e_ yielded a straight line in agreement with the Langmuir isotherm giving the isotherm parameters as presented in Table 3.

**Table 3:** Langmuir and Freundlich isotherms data for adsorption by HCPZA **9**.

Adsorbate	Langmuir isotherm				Freundlich isotherm				Dubinin – Radushkevich isotherm			
	*q*_m_ (mg**·**g^−1^)	*k*_L_ (L**·**mg^−1^)	*RL*	*R* ^*2*^	1/*n*	*n*	*K*_f_ (mg**·**g^−1^)	*R* ^*2*^	q_d_ (mg/g)	B_D_ (mol^2^/kJ)	E (*KJ*/*mol*)	*R* ^*2*^
MB	14.9	0.03	0.02	0.9703	1.12	0.89	13.5	0.9799	42	0.013	6.2	0.9867
Hg	31.5	0.35	0.04	0.9907	1.01	0.99	29.5	0.9980	53	0.009	7.5	0.9878
MB mix	22.71	0.08	0.06	0.9981	2.08	0.48	23.5	0.9987	46	0.023	4.6	0.9988
Hg mix	35.0	0.45	0.04	0.9658	1.96	0.51	38.0	0.9899	56	0.008	7.9	0.9869

The values of the Langmuir constants *q*_m_ and *k*_L_ were calculated from the slope and intercept of the plot, and are given in Table 3. From the *R* ^*2*^ values in the data, it is apparent that the adsorption fitted relatively well with the Langmuir Isotherm model. The characteristic parameter of the Langmuir isotherm is demonstrated in terms of the dimensionless equilibrium parameter *R*_L_, also defined as the separation factor by Weber and Chakkravorti^26, 27^.

6 $${R}_{L}=\,\frac{1}{1+\,{K}_{L}\,{C}_{o}}$$

where C_o_ is the initial solute concentration. The value *R*_L_ gives an indication of the type of the isotherm and the nature of the adsorption process. It indicates whether the adsorption nature is either unfavorable (*R*_L_ > 1), linear (*R*_L_ = 1), favorable (0 < *R*_L_ < 1) or irreversible (*R*_L_ = 0). From the data calculated and presented in Table 3, the *R*_L_ values between zero and 1 indicate the favorable nature of the adsorption^28^. Considering the interactions between the adsorbed molecules, the Freundlich model is utilized to describe the adsorption characteristic on the heterogeneous adsorbent surface, using the empirical equation:

7 $${q}_{{\rm{e}}}={{\rm{K}}}_{{\rm{f}}}\,{C}_{e}^{\frac{1}{n}}$$

where *k*_F_ (mg**·**g^−1^) is the Freundlich isotherm constant indicating adsorption capacity and *n* is the adsorption intensity while 1/n is a function of the strength of the adsorption, *C*_e_ is the equilibrium concentration of adsorbate (mg**·**L^−1^) and *q*_e_ is the amount of adsorbate per adsorbent at equilibrium (mg**·**g^−1^). The logarithmic form of Freundlich is defined as:

8 $$\mathrm{ln}\,{q}_{{\rm{e}}}={{\rm{lnK}}}_{{\rm{f}}}+\frac{1}{{\rm{n}}}{\mathrm{lnC}}_{{\rm{e}}}$$

From the plot of ln *q*_e_ versus ln C_e_, *K*_F_ and n were calculated as shown in Table 3. The *n* values give an indication of the favorability of the adsorption process. The value of *n* > 1 represents a favorable adsorption. A value of 1/*n* < 1 indicates a normal adsorption while 1/*n* > 1 indicates a cooperative adsorption. In this study, the value of 1/*n* is less than 1 indicating a favorable adsorption process of the dye and Hg on the polymer. The *k*_F_ and *R* ^*2*^ values in Table 3 show that the Freundlich model gives the best fit for the four systems studied.

The data were fitted to the Dubinin-Radushkevich (D-R) isotherm model applied to the data to deduce the heterogeneity of the surface energies of adsorption and the characteristic porosity of the polymer. The linear form is:

9 $$\mathrm{ln}\,{q}_{{\rm{e}}}=\,\mathrm{ln}\,{q}_{{\rm{D}}}\mbox{--}{B}_{{\rm{D}}}{[{\rm{RTln}}(1+1/{C}_{{\rm{e}}})]}^{2}$$

The apparent energy of adsorption was calculated as:

10 $$E=1/{(2{B}_{{\rm{D}}})}^{1/2}$$

where *q*_D_ is the D-R constant indicating the theoretical saturation capacity and B_D_ is a constant or the mean free energy of adsorption, R is the ideal gas constant, (8.314 J/mol K), T (K) is the temperature of adsorption, and E is the mean free energy of adsorption per molecule of the adsorbate when transferred to the surface of the solid from infinity in solution. From the plot of ln*q*_e_ against [RTln(1 + 1/C_e_)]^2^, the constants q_D_ and B_D_ were calculated from the intercept and the slope and E was calculated from the obtained B_D_.

If the value of E lies between 8 and 16 kJ/mol the sorption process is a chemisorptions process, while values below 8 kJ/mol indicate a physical adsorption process^29, 30^. The value of the apparent energy of adsorption in Table 3 indicated a tendency towards chemisorption for the Hg(II) adsorption (E ≈ 8 kJ/mol) and physisorption for the MB.

### Characterization of the polymer after sorption

**Figure 9:**
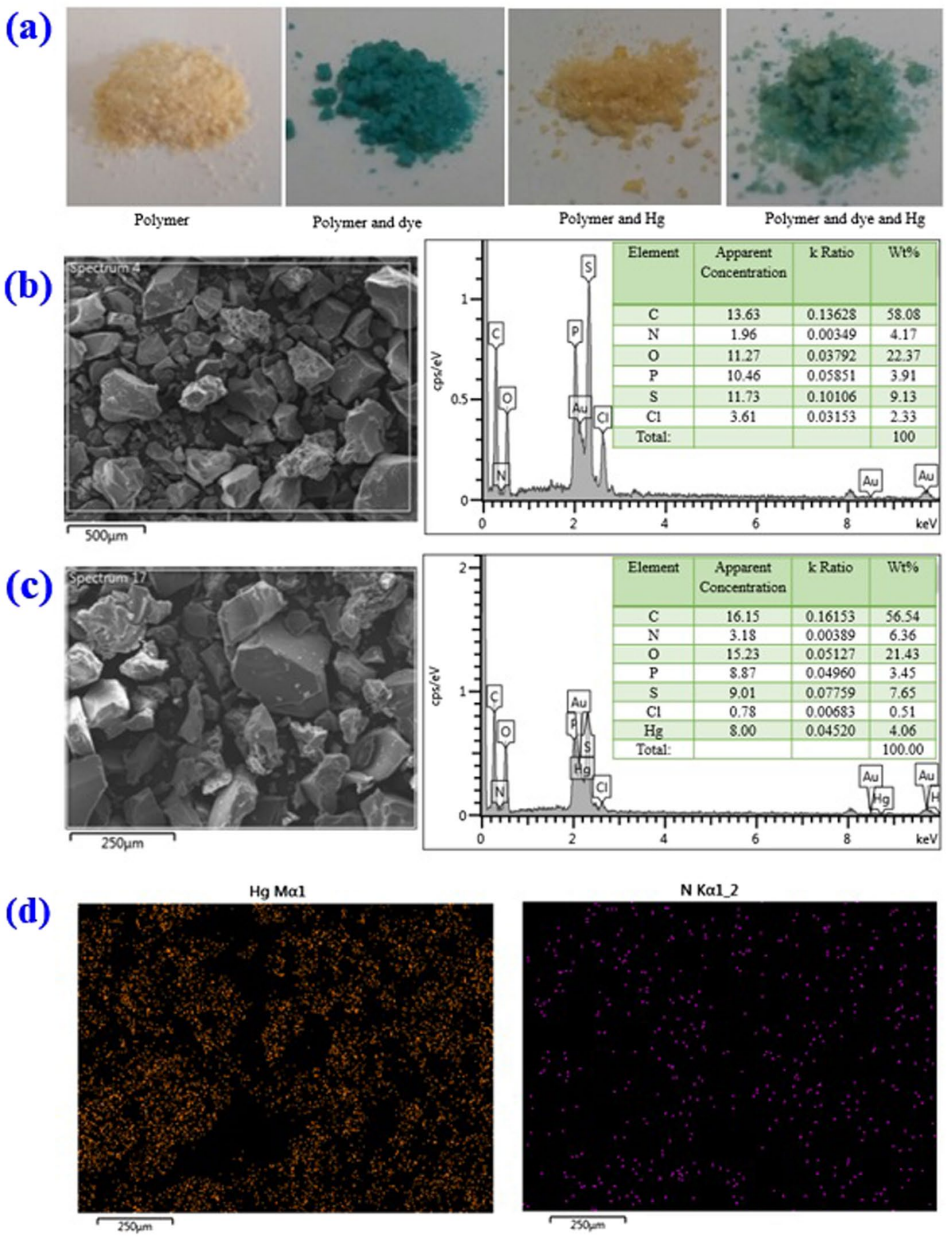
(**a**) Photo of the resin, dye-loaded resin, Hg-loaded polymer, and dye and Hg-loaded polymer; (**b**) SEM image and EDX spectrum of the prepared polymer; (**c**) SEM image and EDX spectrum of the dye and the Hg-loaded resin; (**d**) X-ray absorption elemental mapping of the mercury and nitrogen in the dye and Hg-loaded resin.

The analysis of the adsorbate loaded polymer was conducted using SEM, EDX, IR and XPS. The polymer was collected after adoption by separation. The photos depicted in Fig. 9 show the change in the resin color because of the interaction between the dye or Hg with the adsorbent. Comparing the SEM/EDX results of the resin before and after the adsorption of dye and Hg, one can notice the presence of the mercury in the EDX spectrum at 21 and 88 keV. The amount of the nitrogen was also increased, indicating the presence of the dye on the surface as methylene blue contains nitrogen in the structure. The mercury and nitrogen mapping give some indication of the adsorbate distribution in the resin. The data confirm the possible binding of Hg(II) to the surface of the polymer.

**Figure 10:**
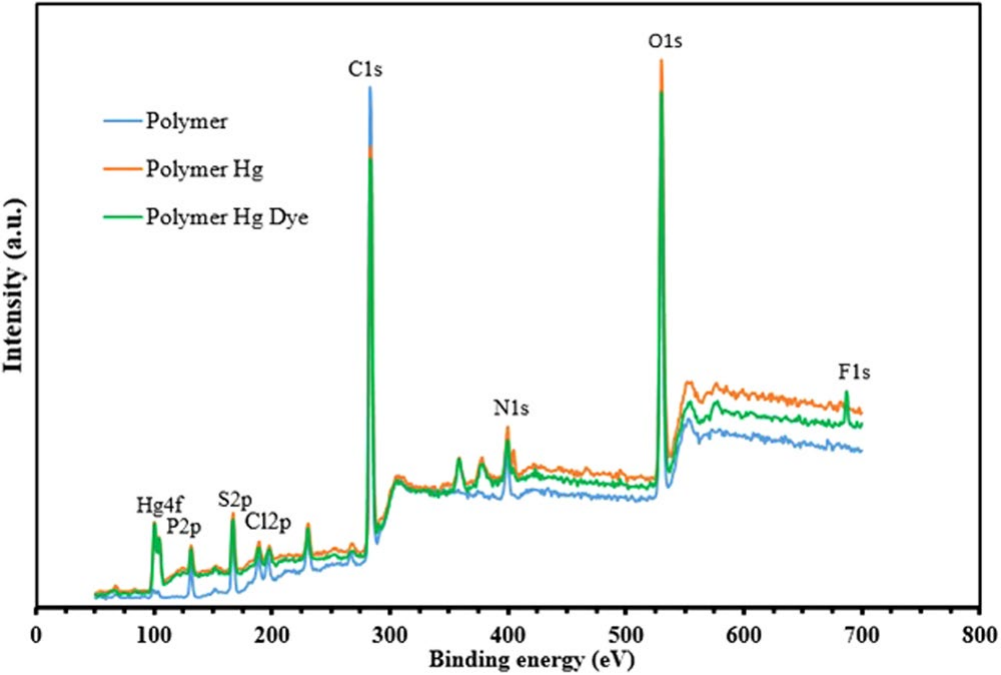
X-ray photoelectron spectroscopy (XPS) spectra of the pristine polymer sample, Hg(II)-loaded polymer and Hg-dye-loaded polymer, after adsorption.

As depicted in Fig. 10, the mercury immobilization on the surface is examined by an XPS spectra of the resin before and after the adsorption of Hg(II). A new peak at 101 eV corresponding to the Hg (4f) orbital appears after the mercury contact^31, 32^.

In the dye loaded resin, the amplified C=C stretching vibration at 1600 cm^−1^ confirms the adsorption of the dye since both the resin and dye show vibration at 1600 cm^−1^ (*cf*. Fig. 5: a *vs*. c). Comparisons between the spectra of the Hg(II)-loaded resin and the pristine resin indicate a hypsochromic shift and a decrease in some wavenumbers for the bands of the resin, indicating the chelation between the phosphonate groups and the mercury ions (*cf*. Fig. 5a vs. 3d)^33^.

### Immobilization mechanism

**Figure 11:**
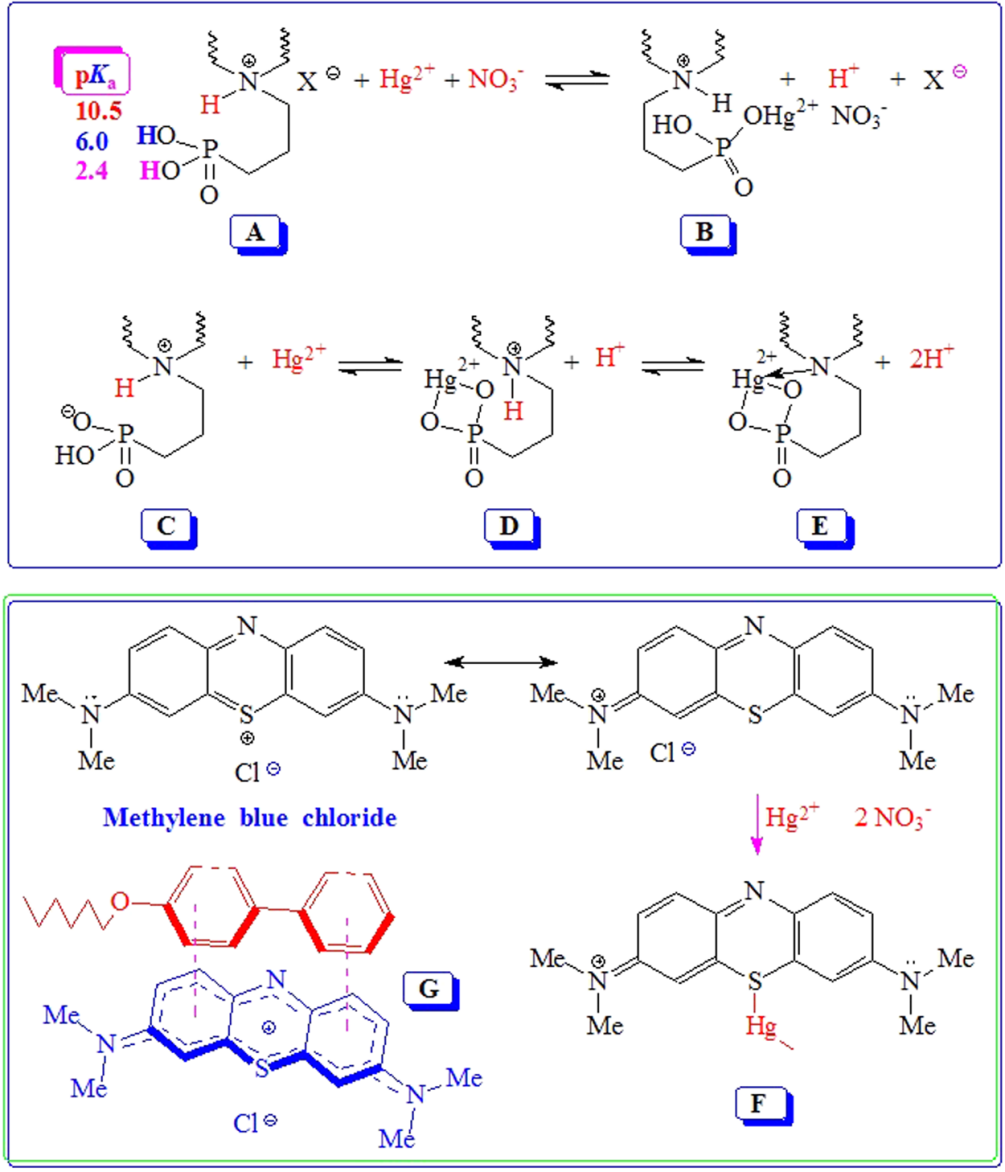
Aminopropylphosphonate as a chelating ligand and Hydrophobic interaction between the MB and the p-phenylphenoxy pendant.

The three p*K*_a_ values for the triportic species **A** are expected to be around 10.5, 6.0 and 2.4 (Fig. 11)^34, 35^. The adsorption capacity of Hg(II) is increased with the increase of the solution pH values, particularly in the pH range of 1.0–5.0. The larger concentrations of H^+^ ions at lower pH values push the A $\rightleftharpoons$ B equilibrium towards the left. With increasing pH values, the formation of **B** is preferred. In the entire pH window, the cross-linker units will have cationic charges which can bind NO_3_^−^. The presence of new bands around 1383 cm^−1^ (Fig. 5d) due to the nitrate group suggests the ability of the resin to act also as an anion-exchanger^36, 37^. At higher pH values, increased negative charge density on the polymer pendants would encourage ion exchange as well as complexation via the bidentate ligand to give species **D**. The chelating functionality of aminopropylphosphonate may also act as a tridentante ligand as depicted in **E** ^38^. It is worth mentioning that sulfone motifs are also known to act as ligands in metal ion complexes^39, 40^. The study at pH > 5 was avoided since the uptake of Hg(II) cannot be attributed solely to the interaction of the Hg(II) ions with the active sites on the resin. The formation of insoluble Hg(OH)_2_ would lead to erroneous data on the adsorption^41^.

MB is a weak basic cationic dye with a p*K*_b_ of 10.2. While the dye is soluble in water, it can also display hydrophobic interaction because it contains two −N(CH_3_)_2_ groups. The attractive interactions between the MB molecules may lead to possible multilayer adsorption. Owing to resonance, the highly dispersed positive charge in MB is expected to have weak ionic/electrostatic interaction with anionic −PO_3_H^−^ motifs in the resin. Hg(II) can form a bond with MB as depicted in **F** which co-adsorbs on the resin surface^18^. The adsorption of MB may well be augmented *via* hydrophobic interaction and π−π stacking as depicted in **G** (Fig. 11)^34^.

### Regeneration and treatment of real wastewater samples

The regeneration tests were conducted by using 0.1 M HCl for Hg(II) and acetone for methylene blue. The polymer has advantageous properties such as active sites, high adsorption capacity, high reusability, ease-of-use, and the fact that it is cost-effective. The resin has demonstrated remarkable efficiency in removing toxic Hg(II) ions and methylene blue from waters even after 3 cycles with ±3% changes. Thus, the results suggested the next step of testing the resin with the real sample. Industrial wastewater samples were used to study the effect of the real water matrix and to evaluate the practical application of the resin. The sample was spiked with 10000 (µg L^−1^) Hg(II) and 1 µM dye, and then treated with the polymer under the optimum conditions. The dye concentration was analyzed using the UV-vis spectrophotometer. Table 4 presents the analysis of the wastewater sample. The % removals are remarkable; the resin captured efficiently, not only the metal ions, but also As, suggesting its efficiency as an anion exchanger as discussed earlier in the case of NO_3_^−^ (*vide supra*). It is indeed pleasing to see the complete simultaneous removal of the dye. This indicates the high efficiency and capability of the resin to be regarded as a potential highly efficient adsorbent and a renewable adsorbent for Hg(II) ions and methylene blue from aqueous solutions.

**Table 4:** Comparison of Hg(II) and dye concentrations in the wastewater sample before and after treatment with the polymer.

Metal	Original sample (μg L^−1^)	Original sample *spiked* with 10000 (μg L^−1^) Hg(II) and 1 μM dye; then treated with the polymer	Removal (%)
Hg	10.85	88.23	99
Pb	14.45	0.56	96
Co	0.37	0.15	60
Cu	758.0	312.2	59
As	6.52	1.25	81
Mo	29.39	1.98	93
Cd	3.18	<MDL	≈100
Dye	1 µM	<MDL	≈100

MDL: the method detection limit

The maximum percentage removal (%) of Hg(II) in the presence of MB dye, i.e. simultaneous adsorption, using the current resin was 99%, which was found to be comparable with other adsorbents in the literature. For example, 94.5% of mercury was reported to be removed using xanthate functionalized magnetic graphene oxide^42^, 94.0% of mercury was reported to be removed using magnetic graphene oxide^43^, 98.4% of mercury was reported to be removed using magnetic polydopamine–chitosan^44^, while 99.5% of mercury was reported to be removed using glutamine modified chitosan magnetic composite^6^.

In addition, there are some other important factors for comparing the sorbent materials, such as regenerability and cost. The material can be recycled several times as discussed above. With respect to the cost of producing the adsorbent, considering the amount of the materials used for 1 Kg production, the net price is estimated as 15 USD. However, cost information is rarely reported, and the expense of each adsorbent varies depending on availability and the degree of processing required. Note that the cost of the adsorbents in the previously mentioned literature was not reported.

## Conclusions

The present study was focused on the synthesis, chemical, morphological and thermal characterization of a new novel resin. We reported on the investigation of the adsorption potential of newly developed resin in the single and simultaneous exclusion of Hg(II) ions and methylene blue from aqueous solutions. The resin showed a good adsorption performance with a high Langmuir monolayer adsorption capacity at pH 5 at 24 °C. The kinetic assessments revealed that the adsorption process of Hg(II) and methylene blue onto the polymer was facilitated via the second-order kinetic mechanism with an *R* ^2^ value of >0.99 for all of the studied concentrations. The initial concentration, temperature, and pH, as well as the surface-active sites, all contribute to the adsorption efficiency. The regeneration tests were achieved by using 0.1 M HCl for Hg(II) and acetone for methylene blue. In addition to being cost-effective, the resin has advantageous properties such as active sites, high adsorption capacity, high reusability, and ease-of-use, in addition to being cost-effective. The polymer has demonstrated remarkable efficiency in the simultaneous removal of toxic Hg(II) ions and methylene blue from aqueous systems.
